# Diagnostic Accuracy of Physical Examination and History Taking in Traumatic Rib Fracture; A Single Center Experience 

**DOI:** 10.30476/BEAT.2020.46451

**Published:** 2020-04

**Authors:** Navid Kalani, Seyed Reza Habibzadeh, Roya Ghahremanizadeh, Ayoub Tavakolian, Naser Hatami, Saeed Barazandehpour, Samaneh Abiri

**Affiliations:** 1 *Anesthesiology, Critical Care and Pain Management Research Center, Jahrom University of Medical Sciences, Jahrom, Iran.*; 2 *Research Center for Social Determinants of Health, Jahrom University of Medical Sciences, Jahrom, Iran.*; 3 *Department of Emergency Medicine, Faculty of Medicine, Mashhad University of Medical Sciences, Mashhad, Iran.*; 4 *Department of Emergency Medicine, Mashhad University of Medical Sciences, Mashhad, Iran.*; 5 *Department of Emergency Medicine, Sabzevar University of Medical Sciences, Sabzevar, Iran.*; 6 *Student Research Committee, Jahrom University of Medical Sciences, Jahrom, Iran.*; 7 *Department of Emergency Medicine, Kerman University of Medical Sciences, Kerman, Iran.*; 8 *Department of Emergency Medicine, Jahrom University of Medical Sciences, Jahrom, Iran.*

**Keywords:** Physical examination, Rib fracture, Sensitivity, Specificity

## Abstract

**Objective:**

To evaluate the diagnostic accuracy of history taking and physical examination in the patients with traumatic rib fractures.

**Methods:**

In a cross-sectional study, all patients with multiple traumas who referred to the emergency department were evaluated for the mechanism of injury, chief complaints, vital signs and oxygen saturation. History taking and physical examination were performed according to Barbara Bates reference. Fracture was diagnosed based on chest x-ray results and CT scan, if needed. The results were analyzed by receiver operating characteristic (ROC) curves and area under the curve (AUC) analysis.

**Results:**

Isolated rib fractures of thoracic bones were found in 8 out of 99 subjects with mean age of 33.4±19.43 years. In the sensitivity analysis of history taking and physical exam tests, the highest sensitivity was chest tenderness and deformity with 100% sensitivity for each one and the lowest was for the dyspnea with 28.10%; however, the highest sensitivity was for dyspnea with 62.50% sensitivity; and pulmonary hearing aid and chest deformity were not specific (0%). For heart rate, AUC analysis was significant. Heart rate above 80/min was associated with 87.5% sensitivity and 62.5% specificity for rib fractures.

**Conclusion:**

Proper and physical examination and history taking can help to detect rib fractures with high sensitivity and specificity denoting to the importance of the issue; while, radiographic or surgical approval is required to diagnose rib fractures.

## Introduction

Currently, trauma is the leading cause of death, hospitalization, and disability for all age groups, and that is why trauma victims are more likely killed more than other diseases. Chest trauma alone accounts for 45% of all trauma-related deaths. The key to diagnose chest injuries is having a strong mindset about the likelihood of thoracic trauma in injured patients, based on accurate patient history [1]. The severity and type of mechanism that cause the trauma is obtained and a large percentage of injuries can be diagnosed by simple paraclinical techniques, especially by plain chest radiography [2].

However, in less than 15% of the injuries to the chest, emergency surgery is needed. In other injuries, only supportive care and early treatment are sufficient. Many of these injuries are considered moderate in severity and require only a limited number of surgical interventions, and there is little doubt that such injuries are of particular importance [3]. Careful follow-up of these patients is essential and very helpful in identifying cases that require the treatment of operations [4]. Thoracic trauma is recognized as an important problem and its therapeutic significance is clear [5]. Among the fractures during the chest trauma, fractures of the ribs are not common in infants due to chest flexion, but are very common in the elderly. Most rib fractures occur in the middle part of the rib [6]. Upper fractures can be associated with aortic and tracheal injuries and lower rib fractures with damage to the intra-abdominal organs, including the kidney, spleen, and liver [7]. Some evidences suggest that there is a direct relationship between age, increased number of damaged ribs, and mortality [8]. In this study, we have evaluated the sensitivity and specificity of history taking and physical examination in the diagnosis of isolated rib fractures. 

## Materials and Methods

*Study population*

In this cross-sectional study with ethical approval code of IR.JUMS.REC.1396.102 from Jahrom University of Medical Sciences, Jahrom, Iran, all patients with multiple trauma referred to the hospital emergency department during the 10-month period were assessed. Patients with indications for chest x-ray (blunt trauma patients) were first examined by a physician and the mechanism of injury, chief complaints, vital signs, and oxygen saturation were recorded. Prior to chest x-ray, all patients underwent linear probe ultrasound by the emergency department and the results were recorded. 

*Study protocol*

Chest radiographs were obtained from all patients. Chest computed tomography was performed based on the indication. Radiographic evaluation was performed on the basis of a CT scan. Radiology images were reported by 2 radiologists and the degree of agreement was calculated. Fractures were diagnosed based on chest x-ray findings and CT scan, if needed. Exclusion criteria were as follows: Patients who were traumatized for more than 6 hours, patients who died, multiple traumatic patients with unstable vital signs, intra-abdominal bleeding, and fractures other than the rib in the chest bones. Finally, 99 patients were included in the study. History taking and physical examination was performed based on "Bates' Guide to Physical Examination and History Taking" for distractor pain, dyspnea, vital sinus, skin abrasion, chest tenderness, pulmonary crystallization, thoracic deformity, abdominal tenderness, and reduced lung sounds [9]. Information forms were coded and demographic information was confidential to the researcher. 

*Statistical analysis*

The information obtained from the patients was analyzed using SPSS software (Version 16, Chicago, IL, USA). Because chest x-ray examination is the gold standard in rib diagnosis, we measured sensitivity, specificity, positive predictive value, negative predictive value, and accuracy associated with radiographic findings. The results were analyzed using receiver operating characteristic (ROC) curves and area under the curve (AUC) analysis.

## Results

Ninety-nine patients with mean age of 33.4±19.43 years were studied. There were 58 male and 41 female subjects. Isolated rib fractures of chest bones were seen in 8 participants. The characteristics of the studied subjects were listed in Table 1. According to Table 2, among the sensitivity and specificity of each of the vital signs tests, only the heart rate AUC analysis was significant (*p*=0.016). Heart rate above 80/min was associated with 87.5% sensitivity and 62.5% specificity for rib fractures.

According to Table 3 in assessment of the specificity of physical exam tests, the highest sensitivity was related to chest tenderness and deformity with 100% sensitivity for each one and lowest for dyspnea with 28.10%; however, the highest specificity was for dyspnea with 62.50% sensitivity; chest tenderness, cryptography in pulmonary hearing aid, and chest deformity were not specific (0.0%).

**Table 1:** Characteristics of study participants

**Multiple trauma patients (n=99)**	**Variable **	
33.4 (19.43)	Age, mean (SD)	
58 (60.4)	Sex (male), n (%)	
11 (11.11)	Motorcycle accident	Trauma mechanism, n (%)
43 (43.43)	Car accident	
10 (10.10)	Falling down from higher than 3 meters	
6 (6.6)	Falling down without height	
12 (12.12)	Pedestrian	
17 (17.17)	Others	
8 (8.08)	Rib	Fractures, n (%)
0 (0)	Scapula	
0 (0)	Sternum	
0 (0)	Clavicle	
0 (0)	Vertebra	

**Table 2:** Sensitivity and specificity of vital sinus in relation to rib fracture

**Specificity (%)**	**Sensitivity (%)**	**Cut-off**	**P value**	**AUC**	**Variable**
62.5	87.5	79	0.016	0.759	Heart rate
98.8	87.5	93	0.913	0.488	BPS
97.5	87.5	53	0.701	0.459	BPD
80	50	14	0.425	0.414	Respiratory rate
98.8	87.5	94	0.581	0.441	O_2_
95	87.5	4	0.606	0.445	GCS

**AUC:** Area Under Curve, **BPS: **Blood Pressure Systolic, **BPD:** Blood Pressure Diastolic **GCS: **Glasgow Coma Scale

**Table 3:** Specific sensitivity of history taking and physical exam in the diagnosis of rib fractures

**NPV** ^b^ ** (%)**	**PPV** ^a^ ** (%)**	**Specificity (%)**	**Sensitivity (%)**	**Variable**
0.953	0.111	50.00	71.91	Distracting pain
0.938	0.061	62.50	28.10	Dyspnea
0.987	0.116	37.50	94.38	Scratches on the skin
NA^c^	0.08	0.00	100.00	Chest tenderness
0	0.078	0.00	98.90	Cryptography in pulmonary hearing aid
NA^c^	0.08	0.00	100.00	Chest deformity
0.967	0.106	37.50	85.40	Abdominal tenderness
0.953	0.111	50.00	71.91	Reduce lung sounds

^a^ **PPV:** Positive predictive value; ^b^**NPV: **Negative predictive value; ^c^**NA: **Not applicable

## Discussion

Delay in the diagnosis of musculoskeletal injuries in patients with multiple trauma can lead to functional impairments throughout their lives. Understanding the underlying causes of injuries in multi-trauma patients reduces its incidence and prevents many disabilities and disorders [10]. Complete and accurate physical examinations, and selective x-rays with standard diagnostic protocols can prevent many damages caused by hidden injuries [11]. Serial physical examination during hospitalization and after discharge is also useful in detecting hidden injuries [12]. 

Chest is important because of lung, heart, vascular and tracheal organs because thoracic trauma can cause damage to these organs [13]. In blunt trauma, trauma can cause fractures of the ribs, sternum, lung, heart and aorta. In blunt trauma, although the surface of the skin is healthy, fractures of the ribs can cause various organ's rupture [14]. Signs and symptoms of chest injury include: pain at the site of injury, respiratory exacerbated local pain at the site of injury, shortness of breath, tachycardia, hypotension, cyanosis and etc. [15]. Rib fractures could be clinically diagnosed without imaging based on history and physical examination. Given the benign clinical pattern of single rib fractures, specific rib x-ray series are not usually required [16]. This study showed the incidence of 8.08% of rib fractures in multiple trauma patients. Sirmali *et al*.’s study showed the incidence of 6-12% of isolated rib fractures in traumatic patients. We have focused on isolated rib fractures to elicit the probable effects of other fractures in chest, to show the importance of the physical examination [17].

Our study showed that chest tenderness and deformity had the most sensitivity in our evaluated physical exam tests. As well as our study, Rutherford *et al.* mentioned that rib fractures are usually detected by x-rays in the chest along with a physical examination of point tenderness and palpable deformity [18]. The limitations of this study were as follows: not evaluating the severity of trauma due to the type of injury and its mechanism as well as to determine the number of days of hospitalization and need for artificial respiratory system and need for surgical intervention. 

In conclusion, the results of the current study demonstrate proper and timely diagnosis and treatment methods of traumatic rib fractures are important; and history taking and physical exam are in a greater importance in accurate diagnosis of rib fractures. 
